# Short-Term Effects of Sacubitril/valsartan on Echocardiographic Parameters in Dogs With Symptomatic Myxomatous Mitral Valve Disease

**DOI:** 10.3389/fvets.2021.700230

**Published:** 2021-07-27

**Authors:** Nakkawee Saengklub, Prapawadee Pirintr, Thanida Nampimoon, Anusak Kijtawornrat, Narongsak Chaiyabutr

**Affiliations:** ^1^Department of Physiology, Faculty of Pharmacy, Mahidol University, Bangkok, Thailand; ^2^Department of Veterinary Biosciences and Veterinary Public Health, Faculty of Veterinary Medicine, Chiang Mai University, Chiang Mai, Thailand; ^3^Department of Physiology, Faculty of Veterinary Science, Chulalongkorn University, Bangkok, Thailand; ^4^The Thai Red Cross Society, Queen Saovabha Memorial Institute, Bangkok, Thailand

**Keywords:** dog, echocardiography, MMVD, sacubitril/valsartan, symptomatic

## Abstract

**Background and Objective:** Sacubitril/valsartan (SV) is an angiotensin receptor-neprilysin inhibitor that works by inhibiting the neprilysin enzyme as well as blocking angiotensin receptors. The benefits of using SV in congestive heart failure patients has been demonstrated in several clinical trials; however, limited data are available for dogs with heart failure. The aim of this study was to investigate the short-term effects of SV in comparison with ramipril in the standard therapy of symptomatic dogs suffering from myxomatous mitral valve disease (MMVD).

**Methods:** In this prospective, randomized, single-blind study, 21 dogs with MMVD stage C were randomly assigned to received SV (20 mg/kg orally twice a day) or ramipril (0.125 mg/kg, orally once a day) in addition to pimobendan and furosemide. Echocardiography, electrocardiography, blood pressure, N-terminal pro-B-type natriuretic peptide (NT-proBNP), and urinary aldosterone per creatinine ratio were obtained at baseline (D0) and at follow-up (4 weeks).

**Results:** When comparing the percent change from baseline between groups, the left atrium to aortic root ratio (LA/Ao) and left ventricular internal diameter diastole normalized to body weight (LVIDDN) were significantly reduced in the SV group (*P* < 0.001 and *P* < 0.01, respectively). The end-diastolic volume index (EDVI), end-systolic volume index (ESVI), and stroke volume were lower in the SV group (*P* < 0.001, *P* < 0.05, and *P* < 0.01, respectively). No changes were observed between groups for NTproBNP, blood pressure, ECG parameters, and urinary aldosterone per creatinine ratio.

**Conclusion:** The current study suggested that the short-term effects of SV can reverse myocardial remodeling, as inferred from several echocardiographic indices (i.e., the reduction in LA/Ao, LVIDDN, EDVI and ESVI) in dogs with MMVD stage C. These findings would support the use of SV in clinically symptomatic heart failure in dogs.

## Introduction

Myxomatous mitral valve disease (MMVD) is the most commonly diagnosed cardiovascular disease in dogs, comprising approximately two-thirds of all cardiac cases (1, 2). Typically, MMVD progresses from mild to severe, related to the magnitude of valvular insufficiency and volume overload. Chronic activation of the sympathetic nervous system (SNS) and renin angiotensin aldosterone system (RAAS) promotes and perpetuates congestive heart failure (CHF). CHF results in high mortality and low quality of life in severely affected dogs (3, 4). Angiotensin converting enzyme inhibitors (ACEi) are indicated in MMVD dogs with CHF (5). Although ACEi have been extensively studied in the chronic management of MMVD in dogs, their efficacy and safety in acute CHF is less clear (6).

A previous study by Mangiafico et al. (7) suggests that the natriuretic peptide (NP) system, which mediates beneficial cardiorenal effects, is also impaired in heart failure. In humans, the rate of degradation of NP is increased with heart failure through the activation of the neprilysin enzyme (8). Enhancing the activity of NP may offer a therapeutic benefit, particularly combined with RAAS inhibition (9). Sacubitril/valsartan (SV), a first-in-class angiotensin receptor neprilysin inhibitor, targets neurohormonal systems (i.e., RAAS and NP) by inhibiting neprilysin to prevent natriuretic peptide degradation, while concomitantly blocking the angiotensin receptor. Recently, the approval of SV by the U.S. Food and Drug Administration for the treatment of heart failure was based on results from the PARADIGM-HF clinical trial (10). The drug has been shown to significantly reduce the rate of cardiovascular death and hospitalization in patients with heart failure compared with enalapril (11). In addition, the Pharmacological Reduction of Functional, Ischemic Mitral Regurgitation (PRIME) study demonstrated that the small changes in left ventricular volume induced by SV resulted in a reduction in functional mitral regurgitation (12).

In veterinary medicine, plasma natriuretic peptides increase with progressively increasing severity of MMVD and the level of plasma natriuretic peptides is associated with mortality rate (13). The pharmacokinetics of SV have been studied in healthy dogs (14, 15); however, there is limited data of SV in HF dogs especially in the management of clinically symptomatic dogs suffering from MMVD. Therefore, the main aim of this study was to compare the efficacy of SV on reverse remodeling in naturally occurring symptomatic MMVD dogs with ramipril used in the standard therapy.

## Materials and Methods

### Animals

The main study was a prospective, randomized, single-blind study. The experimental protocol was approved by the Institutional Animal Care and Use Committee, Faculty of Veterinary Science, Chulalongkorn University, Thailand (protocol no. 1831045). The study was conducted in accordance with the Animals for Scientific Purposes Act, A.D. 2015 and with informed consent of the owners.

### Dose-Finding Pharmacodynamic Study

To determine the optimal dose of SV to be used in MMVD dogs, we studied SV pharmacodynamics through blood pressure (BP), heart rate (HR), and myocardial oxygen consumption (MVO_2_) inferred from the rate pressure product (RPP). Beagle dogs with MMVD stage B1 (*n* = 2) and stage B2 (*n* = 2) were randomized to receive a single oral dose of either placebo (lactose powder packed in a capsule) or SV (5, 10, or 20 mg/kg; Entresto, Novartis Pharmaceuticals, Basel, Switzerland) with a Latin square design. The SV doses were based on the drug composition of SV in the tablets and the dose of valsartan used in dogs (16). On the experimental day, dogs were allowed to become acclimatized to the clinical environment for at least 15 min. Dogs were gently restrained in right lateral recumbency. At baseline and after dosing, the arterial BP and HR were recorded using an oscillometric device (petMAP, CardioCommand, Inc., Tampa, FL, U.S.A.) placed at the left forelimb upon the median artery between the elbow and the carpal pad. All parameters were obtained hourly at baseline (before dosing), 1–6, 12, and 24 h after dosing. Five consecutive measurements of BP and HR were obtained at each timepoint and the mean of three consistent BP and HR was used as an average of those parameters (17). Due to its short elimination half-life, the washout period between each treatment was 7 d.

### Inclusion and Exclusion Criteria for Dogs in the Main Study

In order to be enrolled into the main study, dogs had to be ≥8 y of age with a body weight of ≥2 kg and ≤ 15 kg and had to be in MMVD stage C according to the Consensus Statements of the American College of Veterinary Internal Medicine (17). Each dog had to show all of the following criteria: mitral regurgitation on color Doppler interrogation, holosystolic murmur grade ≥4/6, a left atrial-to-aortic root ratio (LA/Ao) ≥1.6, left ventricular internal diastole diameter normalized to body weight (LVIDDN) ≥1.7 cm, and a vertebral heart score >10.5 vertebral body units. Upon enrollment, each dog had to have clinical signs of CHF including coughing, pulmonary congestion, or edema as shown on thoracic radiograph, dyspnea but not too severe, exercise intolerance, weakness, or syncope. All enrolled dogs that met the inclusion criteria must also be newly diagnosed and never been on treatments with pimobendan or diuretics. Dogs were excluded from the study if they had systemic disease of non-cardiac origin, cardiac arrhythmias, pulmonary arterial hypertension determined by RV: RA pressure gradient >65 mmHg, or congenital heart disease. Dogs were excluded if they had been treated with other medications for CHF within 2 months before entering the study.

### Study Procedures

All animals underwent physical examination, echocardiography, electrocardiography, thoracic radiograph, arterial BP measurement, routine hematology, and routine serum chemistry profiles (i.e., creatinine, blood urea nitrogen, alanine aminotransferase, aspartate transaminase, alkaline phosphatase) before enrolment into the study; these were repeated again at the end of the study (4 weeks after treatment). In addition, blood collection for evaluation of N-terminal pro-B-type natriuretic peptide (NT-proBNP) concentrations and urine collection by cystocentesis for urine aldosterone per creatinine ratio as well as urinalysis were also performed before and after treatment. The sonographers who handled the cases were blinded to treatment allocation. The principal investigator (NS) held the blinding code and randomly allocated each dog to either the ramipril or the SV treatment. SV (49/51 and 97/103 mg/tablet) was given orally at a dose of 20 mg/kg, twice a day. Ramipril (vasotop^®^, Intervet GmbH, Vienna, Austria) was given orally at a dose of 0.125 mg/kg once a day. In addition, all dogs in both groups were given pimobendan (0.25–0.3 mg/kg, twice a day) and furosemide (1.2–1.5 mg/kg, twice a day). Owners were instructed to maintain a constant type and timing of feeding throughout the entire 4 weeks of the study.

Echocardiography was performed on all dogs twice (at baseline and 4 weeks after treatment) without sedation using an ultrasound unit (DC-70 X-Insight, Mindray, Shenzhen, China) equipped with P10-4E (3–11.4 MHz) and SP5-1E (1–5 MHz) phased array transducers. Guidelines for the American Society of Echocardiography were followed during all examinations (18). All echocardiography was performed by an experienced veterinarian (AK) blinded to the study. The mitral valve and tricuspid valve structures were assessed using the left apical 4-chamber view. If tricuspid regurgitation was present, the instantaneous peak systolic tricuspid regurgitant velocity was measured using continuous-wave Doppler interrogation and using a modified Bernoulli equation to calculate the regurgitant velocity. The LA/Ao, LVIDDN, and left ventricular internal diameter systole normalized by body weight (LVIDSN), and shortening fraction (SF) were obtained *via* the right parasternal short-axis view and M-mode, as previously described (19). End-diastolic volume (EDV), end-systolic volume (ESV), and ejection fraction (EF) were determined using a modified Simpson's Method of Discs (20) on the right parasternal long axis 4-chamber view. The EDV and ESV were indexed to body surface area (i.e., EDVI and ESVI, respectively). The quantification of MR jet (%) was obtained by using Doppler echocardiography from left apical 4 chamber view. The area of mosaic color observed during systole inside the left atrium was measured and compared with the total area of the left atrium as described previously (21).

Standard thoracic radiography (i.e., right lateral recumbency and ventro-dorsal projection) was performed in order to confirm the presence of cardiomegaly and pulmonary congestion and edema at enrollment day. The vertebral heart score (VHS) was used to indicate cardiomegaly (10). Pulmonary congestion and edema were evaluated by the presence of an interstitial or alveolar pattern and concurrent clinical signs.

Standard lead II electrocardiography was obtained while dogs were in right lateral recumbency and all limbs were perpendicular to the long axis of the body. The electrodes were clipped onto the skin of all limbs and connected to an ECG machine (Cardimax FX-7302, Fukuda Denshi, Tokyo, Japan). The tracings were assessed for changes in HR and rhythm. All measurements were made of 6 consecutive cardiac cycles and the mean was used (22). The QT interval was corrected for HR using the van de Water formula (23).

Arterial BP was obtained at baseline and 4 weeks after treatment as previously described in the dose-finding pharmacologic study.

Six to ten milliliters of urine was collected by cystocentesis under ultrasonographic guidance. Two milliliters of urine was used to determine concentrations of urine creatinine (U-Cr). The U-Cr was measured using a standard colorimetric assay by the veterinary diagnostic laboratory (Vet Central Lab, Bangkok, Thailand). Four milliliters of urine was centrifuged at 1,500 g for 5 min and the supernatant was collected into polypropylene tubes and stored at −80°C until batch analysis for determination of urine aldosterone concentrations (U-Aldo). The U-Aldo was measured using chemiluminescent immunoassay by the Division of Clinical Chemistry, Department of Pathology, Faculty of Medicine Ramathibodi Hospital. The urine aldosterone per creatinine ratio (UAldo:C) was calculated.

Blood was collected in both EDTA and heparinized tubes. Complete blood count (CBC) was measured using an automated hematology analyzer (ProCyte Dx Hematology Analyzer, IDEXX Laboratories, Inc., Westbrook, ME, U.S.A.). Blood chemistry profiles were measured using a chemistry analyzer (IDEXX Catalyst One, IDEXX Laboratories, Inc., Westbrook, ME, U.S.A.). Additional blood was collected into EDTA tubes for measurement of NT-proBNP. Plasma was separated by centrifugation within 30 min of collection and stored at −80°C ready for batch analysis, according to guidelines established by the manufacturer, and was analyzed using a commercially available assay for measurement of canine NT-proBNP (ABclonal Biotechnology, Woburn, MA, U.S.A.).

### Intraobserver and Interobserver Variability

The method for assessment of the intraobserver variability of the echocardiographic measurement was modified from previous publications (22, 24). In brief, the M-mode echocardiogram from each of 10 dogs were reproduced 10 times and the observer was blinded to the origin of a given image. Mean values, based upon three consecutive cardiac cycles, for left ventricular internal diastole diameter (LVIDd), left ventricular internal systole diameter (LVIDs), and HR were calculated for each dog. The mean, standard deviation of the mean (S.D.), and the coefficient of variation (C = S.D./mean) for all three cardiac cycles were calculated for each dog. Then the mean, S.D. and C for all 10 dogs were calculated, using C as the intraobserver variability. In addition, the mode and difference between the maximum and minimum value were also calculated.

To obtain insight on the relative interobserver differences in echocardiographic measurements of LVIDd and HR, two individuals measured 30 cardiac cycles of M-mode images from three dogs, and the values they obtained were plotted against each other (22, 24). One of the measurers is an extremely well-trained veterinary echocardiographer; the other measurer had no experience with echocardiography and was instructed on how to make the measurements. The agreement between the two measurers was expressed as the R^2^ for the regression line equating LVIDd and HR for the two measurers, and the mean and maximal differences for each parameter were calculated.

### Data Analysis and Statistical Analysis

In the dose-finding pharmacodynamic study, averages of systolic blood pressure (SBP), HR, and RPP were presented as mean ± standard error of the mean (SEM). Differences among groups were determined using two-way ANOVA with repeated measures design followed by Dunnett's test for multiple comparisons with the baseline. In the main study, variables were expressed as mean ± SEM. The normality test was performed using the Shapiro-Wilk test. Percent change from baseline within groups was calculated. Student's *t*-test was used to analyze the differences of percent change from baseline between groups (SV vs. ramipril). A commercial statistical package was used for all statistical analysis and a *P*-value <0.05 was set to indicate statistical significance.

## Results

### Dose-Finding Pharmacologic Study

**Figure 1:**
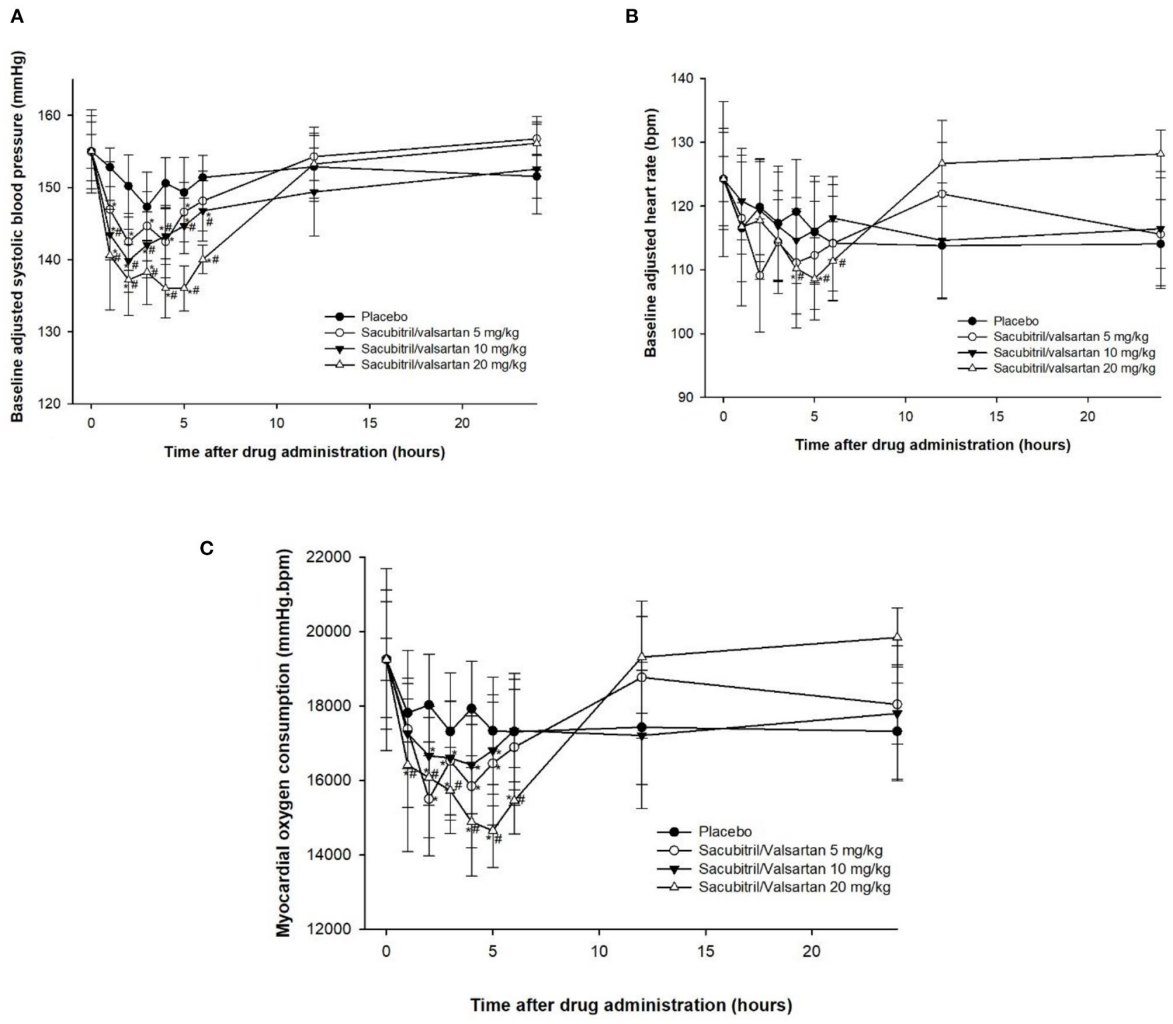
Plots of baseline adjusted systolic blood pressure **(A)**, heart rate **(B)** and rate pressure product **(C)**, an index of myocardial oxygen consumption, against time before (0 h) and after administration (1–24 h) of placebo and sacubitril/valsartan at a dose of 5, 10, and 20 mg/kg, orally in dogs with asymptomatic mitral valve disease stage B1 (*n* = 2) and stage B2 (*n* = 2).

Complete blood count and blood chemical profiles of all four dogs were within normal limits. The thoracic radiograph and echocardiography revealed that all dogs were in ACVIM stage B1 (i.e., mitral valve regurgitation without cardiomegaly) and B2 (mitral valve regurgitation with cardiomegaly but no clinical signs of CHF). No adverse effects were observed in this study. There were no statistically significant differences in SBP at baseline among the groups and within the placebo group among the timepoints (Figure 1A). As expected, increasing SV dosage resulted in progressive SBP reductions. When comparing SV within groups, the reduction in SBP became evident 1 h after dosing, reaching its nadir between 2 and 5 h after treatment. When dogs receiving different doses of SV were compared with the placebo group at the same timepoint, only the 10 and 20 mg/kg groups between 1 and 6 h after dosing were significantly different from the placebo groups (*P* < 0.05). Changes in HR did not parallel the SBP lowering effects (Figure 1B). HR were highly variable among the groups in which only the SV at a dose of 20 mg/kg had significantly lowered HR at 4–5 h after dosing when compared with baseline (*P* < 0.05) and at 4–6 h after dosing when compared with the placebo group at the same timepoint (*P* < 0.05). When considering effects of SV on SBP and HR for each MMVD stage independently (i.e., B1 vs. B2), there was no impact of different stages (B1 vs. B2) on the outcome of the dose finding. RPP was calculated and showed similar tendencies to SBP after drug administration (Figure 1C). However, significant differences among groups of dogs receiving SV when compared with the placebo group at the same timepoint were observed with the 20 mg/kg dose from 1 to 6 h.

### General Characteristics of Dogs in the Main Study

The population for this study included 15 male and 6 female dogs (age range, 6–16 y; and weight range, 2.0–14.9 kg). There were several breeds enrolled in the study including Pomeranian (9), Shih-Tzu (5), Maltese (2), Poodle (2), Chihuahua (2), and Beagle (1). These patients were randomly assigned to SV (*n* = 11) and ramipril (*n* = 10) treatments. No patients were lost to follow-up. At baseline (D0), both groups were similar in terms of age (SV = 11.2 ± 1.00 y vs. ramipril = 11.4 ± 0.87 y, *P* = 0.436), sex, and weight (SV = 4.7 ± 0.49 kg vs. ramipril = 5.8 ± 1.2 kg, *P* = 0.751). The severity of MMVD was similar which was stage C based on ACVIM classification (5). VHS of dogs in both groups was >10.5 and the murmur intensity grade was between 4 and 6/g. In the SV group, dogs were given sacubitril/valsartan at a dose of 20.8 ± 1.44 mg/kg twice a day in addition to standard therapy (pimobendan 0.58 ± 0.04 mg/kg divided into 2 meals and furosemide 1.28 ± 0.08 mg/kg twice a day). In the ramipril group, dogs were given ramipril at a dose of 0.17 ± 0.02 mg/kg once a day in addition to the standard therapy (pimobendan 0.56 ± 0.04 mg/kg divided into 2 meals and furosemide 1.44 ± 0.09 mg/kg twice a day; Table 1).

**Table 1:** Baseline characteristics of the study population dogs with naturally occurring, symptomatic myxomatous mitral valve degeneration.

**Variables**	**MMVD stage C (21)**	
	**Ramipril**	**Sacubitril/valsartan**
	**(*n* = 10)**	**(*n* = 11)**
**Age (y)**	11.4 ± 0.87	11.2 ± 1.00
**Body weight (kg)**	5.8 ± 1.20	4.7 ± 0.49
**Breeds**		
Beagle	1	0
Chihuahua	1	1
Pomeranian	4	5
Poodle	1	1
Shih-Tzu	2	3
Maltese	1	1
**Genders**		
M/Mc/F/Fs	5/2/1/2	5/3/0/3
**Thoracic radiography**		
VHS	10.89 ± 0.07	11.33 ± 0.27
**Murmur intensity**		
Grade 4/5/6	8/2/0	8/3/0
**Dose of medication**		
Pimobendan (mg/kg/day)	0.56 ± 0.04	0.58 ± 0.04
Furosemide (mg/kg)	1.44 ± 0.09	1.28 ± 0.08
Ramipril (mg/kg)	0.17 ± 0.02	-
Sacubitril/valsartan (mg/kg)	-	20.8 ± 1.44

*Data are presented as mean ± standard error mean (SEM). MMVD, myxomatous mitral valve degeneration; n, number; M, male; Mc, castrated male; F, female; Fs, spayed female; VHS, vertebral heart score*.

### Echocardiographic Parameters

**Figure 2:**
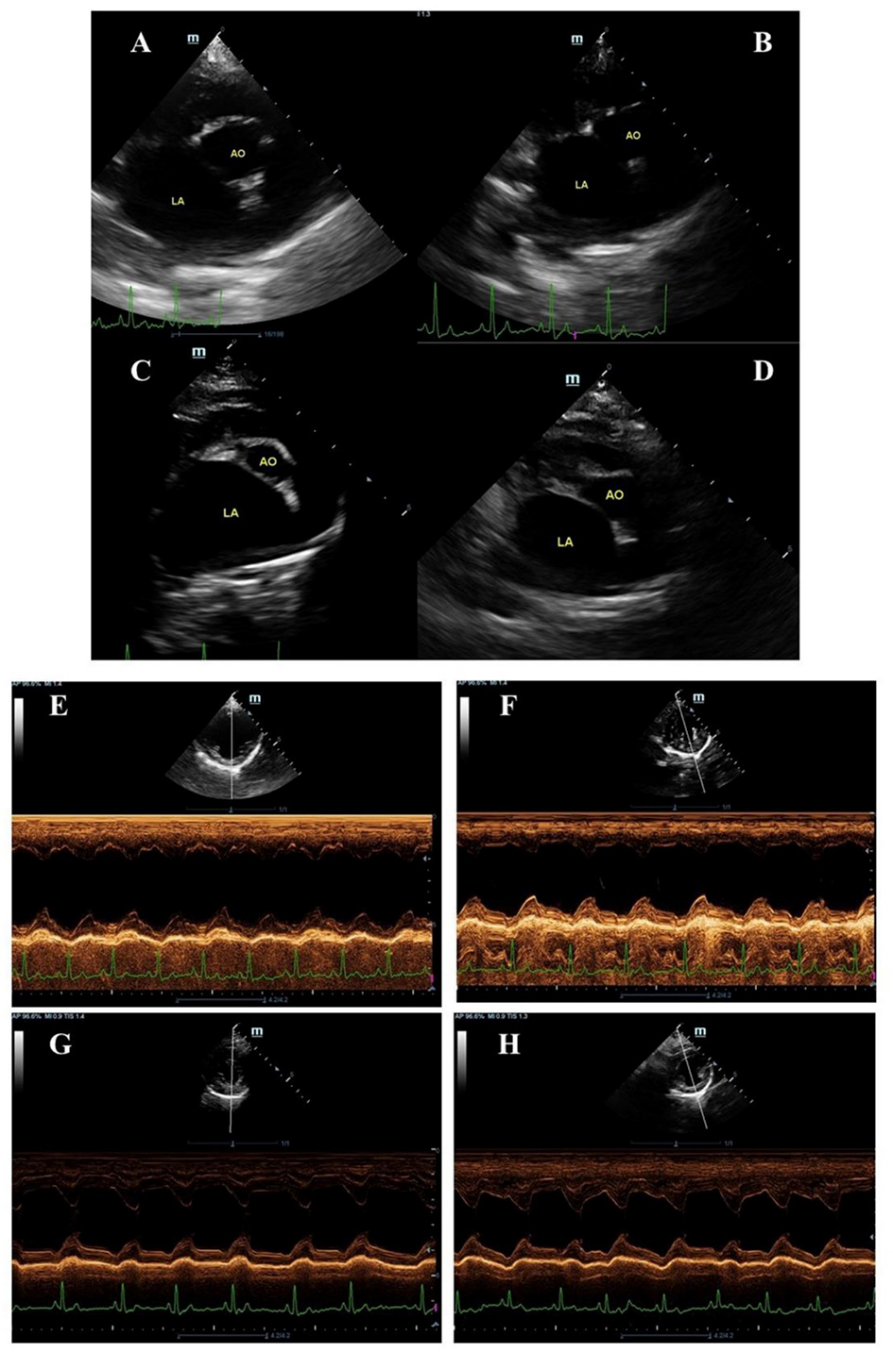
Examples of two-dimension echocardiography of parasternal short axis view at the level of the left atrium and aorta **(A–D)** and M-mode **(E–H)** in symptomatic dogs with myxomatous mitral valve disease stage C before **(A,C,E,G)** and after **(B,D,F,H)** being given ramipril **(A,B,E,F)** sacubitril/valsartan **(C,D,G,H)**. The left atrium to aortic root ratio (LA/Ao) and left ventricular internal diastole diameter normalized to body weight (LVIDDN) were obtained via the right parasternal short-axis view and M-mode as described by Boswood and colleagues (19). The LA/Ao measurement was done by the “Swedish” method (25) while the LVIDDN measurement was done according to (26).

The echocardiographic data of SV and ramipril groups were assessed at baseline (D0) and the follow-up examination was performed at 28 d after initial treatment; data are summarized in Table 2. When comparing the difference from baseline between groups, there were several parameters that changed significantly. LA/Ao changed from 2.31 ± 0.01 to 1.91 ± 0.61 in the SV group and from 2.38 ± 0.13 to 2.26 ± 0.12 in the ramipril group (Figures 2A–D). The percent change from baseline was significantly different between the SV group and the ramipril group (−17.0 ± 1.79 vs. −5.6 ± 1.10, *P* = 0.00002). LVIDDN changed from 1.90 ± 0.08 cm to 1.68 ± 0.07 cm in the SV group and from 1.87 ± 0.12 cm to 1.77 ± 0.08 cm in the ramipril group, and the percent change from baseline was statistically significant (*P* = 0.007; Figures 2E–H). At follow-up, the percent changes from baseline of EDVI and ESVI were significantly lower in the SV group than in the ramipril group (*P* = 0.0006 and *P* = 0.049, respectively). The stroke volume showed a greater reduction from baseline in the SV group when compared with those of the ramipril group (−21.16 ± 3.48% vs. −6.70 ± 2.55%, *P* = 0.002). The percent change from baseline of LVIDSN tended to decrease in the SV group when compared with the ramipril group (*P* = 0.056). There were no significant differences in the changes of FS, EF, CO, HR, and MR jet area between the treatment groups.

**Table 2:** Effects of ramipril and sacubitril/valsartan on echocardiographic parameters in dogs with naturally occurring, symptomatic myxomatous mitral valve degeneration.

**Echocardiographic** **parameters**	**Timepoints** **(weeks)**	**Ramipril** **(*n* = 10)**	**Sacubitril/** **valsartan** **(*n* = 11)**	***P*-value**
LA/Ao	Baseline	2.38 ± 0.13	2.31 ± 0.01	0.347
	4	−5.6 ± 1.10	−17.0 ± 1.79	0.00002
LVIDDN (cm)	Baseline	1.87 ± 0.12	1.90 ± 0.08	0.460
	4	−5.33 ± 1.83	−11.66 ± 1.84	0.007
LVIDSN (cm)	Baseline	0.94 ± 0.05	0.96 ± 0.04	0.459
	4	−3.67 ± 6.35	−15.32 ± 3.36	0.056
FS (%)	Baseline	46.74 ± 1.88	47.03 ± 1.48	0.453
	4	3.47 ± 6.47	7.28 ± 3.12	0.296
EF (%)	Baseline	79.01 ± 2.02	79.20 ± 1.51	0.470
	4	1.58 ± 3.69	4.60 ± 1.82	0.230
EDVI	Baseline	114.3 ± 11.61	120.8 ± 9.95	0.597
	4	−8.33 ± 2.02	−23.65 ± 3.40	0.0006
ESVI	Baseline	24.81 ± 4.60	25.28 ± 3.36	0.504
	4	−2.10 ± 17.58	−33.62 ± 6.61	0.049
SV (mL)	Baseline	28.37 ± 4.53	26.40 ± 2.65	0.916
	4	−6.70 ± 2.55	−21.16 ± 3.48	0.002
CO (L/min)	Baseline	3.83 ± 0.61	3.30 ± 0.36	0.805
	4	−3.64 ± 5.02	−10.28 ± 3.62	0.145
Heart Rate (bpm)	Baseline	139 ± 4.35	133 ± 8.97	0.276
	4	2.17 ± 6.68	12.18 ± 9.80	0.205
Jet area (%)	Baseline	87.79 ± 2.80	87.46 ± 3.04	0.468
	4	−6.05 ± 4.97	−8.99 ± 4.48	0.333

*Values at day baseline are presented as actual number, whereas values at 4 weeks are presented as percent change from baseline. The jet area (%) is the quantification of mitral regurgitation jet by compared the mosaic area with the total left atrial area during systole. Data are presented as mean ± standard error mean (SEM). Differences between groups at the same timepoint were compared using Student's t-test, and values of P < 0.05 were considered significance. LA/Ao, left atrial to aortic ratio; LVIDDN, left ventricular internal diameter diastole normalize; LVIDSN, left ventricular internal diameter systole normalize; FS, fractional shortening; EF, ejection fraction; EDV, end-diastolic volume; DSV, end-systolic volume; CO, cardiac volume*.

### Intraobserver and Interobserver Variability

Measurements of LVIDd, LVIDs, and HR made from each of 3 consecutive cardiac cycles duplicated 10 times for each dog revealed that the means and modes for each dog were virtually identical for all three parameters measured (Supplementary Table 1), and the coefficient of variation of the 10 repeated measurements was small. The greatest coefficient of variation of the 10 measurement was in the LVIDs for dog 1, and this was only 4.96%. The mean coefficients of variation were 1.76% for LVIDd, 3.44% for LVIDs, and 0.94% for HR.

The values for HR were virtually identical (*R*^2^ = 0.9951) between the two measurers, and the values for LVIDd were very close (*R*^2^ = 0.9303). The mean difference in LVIDd between the two measurers was 0.57% (or 0.013 cm) and the maximal difference was 1.69% (or 0.04 cm).

### Other Parameters

In this study, several parameters including electrocardiography, blood pressure, NTproBNP, and UAldo:C were obtained. There was no significant change from baseline for those parameters in group comparisons (Table 3). All ECG tracings demonstrated no significant arrhythmia during the study period in both groups. CBC and blood chemistry profiles obtained at baseline and at follow-up did not demonstrate any clinically abnormal change.

**Table 3:** Effects of ramipril and sacubitril/valsartan on electrocardiographic parameters, blood pressure, and N-terminal pro-B-type natriuretic peptide (NT-proBNP), urine aldosterone per creatinine ratio (UAldo:C) in dogs with naturally occurring, symptomatic myxomatous mitral valve degeneration.

**Parameters**	**Timepoints** **(weeks)**	**Ramipril** **(*n* = 10)**	**Sacubitril/** **valsartan** **(*n* = 11)**	***P*-value**
**Electrocardiography (ms)**				
PQ intervals	Baseline	92.1 ± 4.01	92.0 ± 2.83	0.489
	4	2.06 ± 5.10	−3.51 ± 1.90	0.438
QRS duration	Baseline	62.2 ± 1.33	61.3 ± 1.02	0.303
	4	0.75 ± 1.49	−2.88 ± 1.85	0.074
QT intervals	Baseline	201.0 ± 3.97	198.6 ± 2.48	0.296
	4	−0.97 ± 1.99	−3.89 ± 2.11	0.164
QTc interval	Baseline	248.1 ± 4.58	242.2 ± 2.06	0.307
	4	−1.40 ± 1.81	−3.88 ± 1.90	0.179
RR intervals	Baseline	458.1 ± 24.5	498.1 ± 24.9	0.134
	4	5.45 ± 6.26	3.70 ± 5.35	0.417
**Blood pressure (mmHg)**				
SBP	Baseline	182.4 ± 9.56	182.9 ± 9.71	0.487
	4	4.25 ± 9.85	−4.57 ± 5.27	0.214
DBP	Baseline	96.1 ± 8.84	100.9 ± 5.24	0.315
	4	8.60 ± 14.10	1.17 ± 6.77	0.305
NT-proBNP (pmol/L)	Baseline	669.8 ± 156.3	592.6 ± 195.3	0.769
	4	−117.9 ± 61.1	−119.3 ± 61.8	0.987
UAldo:C (pmol/mol)	Baseline	40.5 ± 22.07	29.1 ± 8.87	0.924
	4	−55.45 ± 45.53	−28.98 ± 38.53	0.627

*Values at day baseline are presented as actual numbers, whereas values at 4 weeks are presented as percent change from baseline. Data are presented as mean ± standard error mean (SEM). Differences between groups at the same timepoint were compared using Student's t-test, and values of P < 0.05 were considered significance. SBP, systolic blood pressure; DBP, diastolic blood pressure*.

## Discussion

Since there is no recommended dose for SV in dogs, a dose finding pharmacodynamic study was conducted. The MMVD dogs with ACVIM stage B1 and B2 were used to evaluate the effect of SV on BP as well as HR and RPP. Based on our results, SV at a dose of 20 mg/kg twice a day was chosen for the main study since the changes in SBP and RPP were more consistent when given to these dogs than other groups (i.e., 5 and 10 mg/kg). Interestingly, the dose of SV used in this study was also supported by a recent study in dogs with cardiomegaly secondary to MMVD (27). SV at a dose of 20 mg/kg orally was also used in a rabbit model of myocardial infarction in which it reduced mean BP at 1–2 h after treatment as well as infarct size (28).

In the main study, dogs with MMVD stage C were recruited. The majority of dogs enrolled in this study were Pomeranian (43%) with an age ranging from 6 to 13 y, which was consistent with our previous studies indicating that small breed dogs are more likely to suffer from MMVD than larger breeds (17, 21).

The main finding of the current study demonstrated for the first time that short-term administration of SV in dogs with MMVD stage C induced a greater extent of reverse myocardial remodeling of both LA and LV than in the ramipril group using in standard therapy (ACEi + pimobendan + diuretic), as indicated by several echocardiographic parameters (i.e., LA/Ao, LVIDDN and LVIDSN). It is known that cardiac remodeling in dogs with MMVD involves changes in cardiac morphology as well as cardiac function. In humans, the remodeling is associated with the risk of cardiovascular events (i.e., hospitalization and death) (29). Therefore, the benefit of reverse myocardial remodeling by SV may yield an improved prognosis for MMVD dogs. The results of the current study were also consistent with a large clinical trial in humans, the PARAMOUNT, in which the left atrial size was significantly decreased in the SV group when compared with the valsartan group (30). Our results also demonstrated that EDVI, ESVI, and SV were also reduced in response to SV treatment compared with those of the ramipril group in the standard therapy, which indicates a reduction in heart size. This was consistent with a previous study in HF patients with reduced EF in which EDVI was reduced and associated with a reduction in NT-proBNP and an increase in EF (29).

The changes in EF and plasma NT-proBNP levels were not significant in our study. This may be because the dogs enrolled in our study were in the early phase of ACVIM stage C; therefore, the EF was still preserved while the NT-proBNP was not extremely high. It is known that when the heart is over stretched (e.g., volume overload from mitral regurgitation), NT-proBNP will be released to promote natriuresis, and the level of NT-proBNP is also associated with the severity of heart failure (13). In the present study, NT-proBNP levels were similar between groups at baseline and the levels after treatment were lower when compared with baseline within the same group, which could be due to the smaller heart and less volume distension after treatment. However, the percent changes after treatment were not significantly different between groups. Increased sample size or a longer duration of the treatment may be needed in order to observe a clear difference.

In heart failure patients with reduced EF, the mitral regurgitant of responders was reduced significantly when compared with non-responders (31). This could be associated with LV remodeling (32) and also explained by a reduction in stroke volume (12). In the current study, the percent change from baseline of MR jet area was reduced significantly after treatment with SV when compared with baseline before SV treatment. However, this percent change did not reach statistical significance when compared the percent change between groups.

Urinary aldosterone per creatinine ratio appears to be a useful biomarker for monitoring aldosterone breakthrough (33). In the current study, UAldo:C was highly variable and did not result in a significant difference between treatment groups. This was consistent with a previous study in healthy dogs, and in dogs with naturally occurring myxomatous mitral valve disease, in which UAldo:C was not significantly different among healthy and MMVD dogs of any stage (i.e., B1, B2 and C). This is due to the fact that UAldo:C is affected by several factors (e.g., breed, sex, age and medications) (34).

This study was also designed to determine the intraobserver variability when the same observer measured the same echocardiographic, M-mode image 10 times from 10 dogs. The intraobserver variability is expressed as the coefficient of variation of the mean of the 10 measurements, as well as the difference between the maximum and minimum value. As indicated in the results, the authors believe that the differences are clinically insignificant, and demonstrate that variability of measurements of echocardiographic parameters by the single well-trained observer is small.

The agreement in values for HR and LVIDd between two measurers is extremely close (*R*^2^ for HR being 0.9951 and for LVIDd being 0.9303). It is important to note that any two measurers would produce similar values (22).

In conclusion, SV is effective at lowering LA/Ao, LVIDDN, EDVI, ESVI, and SV in dogs with naturally occurring symptomatic MMVD stage C, which indicated reverse myocardial remodeling. In addition, the short-term effects of SV in these populations were well-tolerated and they did not show any alteration in hematology, blood chemistry profiles, BP, and ECG parameters.

Despite the benefits of prescription SV being superior to ACEi in the standard therapy of MMVD stage C, there remain potential limitations. First, the study was performed using a small sample size. Although this study demonstrated beneficial effects of clinically relevant dosages of SV in MMVD dogs, a larger sample size is needed to confirm the findings. In addition, further studies should be undertaken to examine long-term effects as well as effects during the different stages of MMVD. The pharmacokinetics of SV were not performed; neither were measurements of SV in the blood at the follow-up period. Finally, the current study was a mechanistic study; therefore, the results do not suggest that ACEi should be replaced with SV in dogs with MMVD stage C.

## Data Availability

The raw data supporting the conclusions of this article will be made available by the authors, without undue reservation.
