# Correction: Short-term effects of sacubitril/valsartan on echocardiographic parameters in dogs with symptomatic myxomatous mitral valve disease

**DOI:** 10.3389/fvets.2026.1832646

**Published:** 2026-04-13

**Authors:** Nakkawee Saengklub, Prapawadee Pirintr, Thanida Nampimoon, Anusak Kijtawornrat, Narongsak Chaiyabutr

**Affiliations:** 1 Department of Physiology, Faculty of Pharmacy, Mahidol University, Bangkok, Thailand; 2 Department of Veterinary Biosciences and Veterinary Public Health, Faculty of Veterinary Medicine, Chiang Mai University, Chiang Mai, Thailand; 3 Department of Physiology, Faculty of Veterinary Science, Chulalongkorn University, Bangkok, Thailand; 4 The Thai Red Cross Society, Queen Saovabha Memorial Institute, Bangkok, Thailand

**Keywords:** dog, echocardiography, MMVD, sacubitril/valsartan, symptomatic

In the published article, the Ethics statement was erroneously given as “The animal study was reviewed and approved by the Institutional Animal Care and Use Committee, Faculty of Veterinary Science, Chulalongkorn University, Thailand (protocol no. 1873045).”

The correct Ethics statement is “The animal study was reviewed and approved by the Institutional Animal Care and Use Committee, Faculty of Veterinary Science, Chulalongkorn University, Thailand (protocol no. 1831045).”

In the published article, there was an error. The Institutional Animal Care and Use Committee (IACUC) protocol number in the Materials and Methods section was incorrectly stated as 1873045 instead of the correct protocol number, 1831045.

A correction has been made to the section Materials and Methods, Animals, First Paragraph:

“The main study was a prospective, randomized, single-blind study. The experimental protocol was approved by the Institutional Animal Care and Use Committee, Faculty of Veterinary Science, Chulalongkorn University, Thailand (protocol no. 1831045).”

The original version of this article has been updated.

