# Supplementary material for: Short-Term Effects of Sacubitril/valsartan on Echocardiographic Parameters in Dogs With Symptomatic Myxomatous Mitral Valve Disease
Source: Front Vet Sci. 2021 Jul 27;8:700230. doi: 10.3389/fvets.2021.700230 (PMC8353078; doi:10.3389/fvets.2021.700230)
Supplement: Supplementary file 1 [file Table_1.DOCX]

**Supplement Table 1.** Measurements of left ventricular internal diastole diameter (LVIDd), left ventricular internal systole diameter (LVIDs), and heart rate (HR) for 10 dogs.

|  | **Mean** | | | **S.D.** | | | **C** | | | | **Mode** | | | **Differences** | | |
| --- | --- | --- | --- | --- | --- | --- | --- | --- | --- | --- | --- | --- | --- | --- | --- | --- |
|  | LVIDd  (cm) | LVIDs  (cm) | HR  (bpm) | LVIDd  (cm) | LVIDs  (cm) | HR  (bpm) | | LVIDd  (cm) | LVIDs  (cm) | HR  (bpm) | LVIDd  (cm) | LVIDs  (cm) | HR  (bpm) | LVIDd  (cm) | LVIDs  (cm) | HR  (bpm) |
| **#1** | 2.34 | 1.08 | 142 | 0.03 | 0.05 | 1.26 | | 1.37 | 4.96 | 0.89 | 2.35 | 1.09 | 142 | 0.11 | 0.20 | 4.00 |
| **#2** | 2.38 | 1.19 | 152 | 0.02 | 0.02 | 1.48 | | 0.97 | 1.77 | 0.97 | 2.37 | 1.17 | 153 | 0.08 | 0.06 | 4.00 |
| **#3** | 2.39 | 1.17 | 147 | 0.02 | 0.03 | 1.48 | | 0.97 | 2.28 | 1.00 | 2.38 | 1.19 | 147 | 0.08 | 0.08 | 5.00 |
| **#4** | 2.45 | 1.21 | 97 | 0.05 | 0.03 | 0.95 | | 1.83 | 2.72 | 0.98 | 2.43 | 1.17 | 97 | 0.14 | 0.09 | 3.00 |
| **#5** | 2.41 | 1.23 | 93 | 0.02 | 0.03 | 0.74 | | 0.87 | 2.70 | 0.79 | 2.39 | 1.23 | 93 | 0.06 | 0.11 | 2.00 |
| **#6** | 2.48 | 1.05 | 119 | 0.02 | 0.05 | 1.07 | | 0.69 | 4.54 | 0.91 | 2.49 | 1.09 | 119 | 0.06 | 0.16 | 3.00 |
| **#7** | 2.34 | 1.19 | 114 | 0.03 | 0.05 | 0.88 | | 1.48 | 4.00 | 0.77 | 2.38 | 1.15 | 114 | 0.12 | 0.15 | 3.00 |
| **#8** | 2.29 | 1.16 | 98 | 0.08 | 0.05 | 0.95 | | 3.33 | 4.46 | 0.97 | 2.37 | 1.17 | 98 | 0.21 | 0.17 | 3.00 |
| **#9** | 2.30 | 1.23 | 130 | 0.07 | 0.03 | 1.43 | | 3.08 | 2.72 | 1.11 | 2.35 | 1.24 | 131 | 0.21 | 0.11 | 3.00 |
| **#10** | 2.29 | 1.16 | 122 | 0.07 | 0.05 | 1.17 | | 3.00 | 4.21 | 0.97 | 2.35 | 1.17 | 122 | 0.21 | 0.17 | 4.00 |
| **Mean** | 2.37 | 1.17 | 121 | 0.04 | 0.04 | 1.14 | | 1.76 | 3.44 | 0.94 | 2.39 | 1.17 | 122 | 0.13 | 0.13 | 3.40 |
| **S.D.** | 0.07 | 0.06 | 21.27 | 0.02 | 0.01 | 0.27 | | 1.01 | 1.12 | 0.10 | 0.04 | 0.05 | 21.55 | 0.06 | 0.05 | 0.84 |

Rows show the statistical parameters of all values obtained from each dog whereas columns show the statistical parameters for all 10 dogs. The mean, standard deviation of the mean (S.D.), and the coefficient of variation (C=S.D./mean) for all 3 cardiac cycles were calculated for each dog. Differences between the maximum and minimum value were also calculated.
